# Responsibility for Sickness

**Published:** 1885-05

**Authors:** 


### Responsibility for Sickness.

A proprietor of a hotel at St. Sebastian, Spain, requires payment for the alleged refurnishing of the apartments occupied by an American gentleman who died of heart disease, on the alleged theory that it was contagious. This is common among Continental hotel proprietors. We have not the slightest doubt that if a guest of a hotel in Paris died from the effects of amputation of a limb, a demand would be made upon his estate to pay for the supposed entire refurnishing of his apartment. This swindling custom reminds us of the advice a gentleman once gave a young lady who was complaining of feeling unwell at one of the leading Paris hotels, to the effect that if she thought she was going to die, she had better go out and get into a third-class cab, as her friends would then only be obliged to pay for the cab; whereas, if she died in the hotel, the cost of its refurnishing would probably be demanded.—*Nat. Hotel Reporter.*

